# Cod Liver Oil in Phthisis

**Published:** 1852-01

**Authors:** 


*On Cod Liver Oil in Phthisis.* By Dr. Walshe.—The following conclusions as to the value of the cod-liver oil are taken from the author's recent work on "Diseases of the Heart and Lungs." He states:

1. That it more rapidly and effectually induces improvement in the general and local symptoms than any other agent.

2. That its power of *curing* the disease is undetermined. I mean here by curing the disease, its power of causing, along with suspension of progress, such change in the organism generally as shall render the lungs less prone to subsequent outbreaks of tubercles, than after suspension occurring under other agencies.

3. That the mean amount of permanency of the good effects of the oil is undetermined.

4. That it relatively produces more marked effects in the third than in the previous stages.

5. That it increases weight in favorable cases with singular speed, and out of all proportion with the actual quantity taken ; that hence it must, in some unknown way, save waste, and render food more readily assimilable.

6. That it sometimes fails to increase weight.

7. That in the great majority of cases where it fails to increase weight, it does little good in other ways.

8. That it does not relieve dyspnœa out of proportion with other symptoms.

9. That the effects traceable to the oil in the most favorable cases are: increase of weight, suspension of colliquative sweats, improved appetite, diminished cough and expectoration, cessation of sickness, with cough, and gradual disappearance of active physical signs.

10. That in some cases it cannot be taken, because it disagrees with the stomach, impairing the appetite (without itself obviously nourishing,) and causing nausea, or because it produces diarrhea.

11. That in the former cases it may be made palatable by association with mineral acid, and in the latter prevented from affecting the bowels by association with astringents.

12. That intra-thoracic inflammations and hemoptysis are contra-indications to its use, but only temporarily so. I have repeatedly given the oil within a day or two after cessation of hemoptysis, without any return taking place.

13. Diarrhea, if depending on chronic peritonitis, or secretive change, or small ulcerations in the ileum, is no contra-indication to the use of the oil ; even the profuse diarrhea caused by the ulceration of the large bowel, is not made worse by it.

14. That the good effects of the oil are *cæteris paribus,* directly as the youth of those using it—a singular fact, which probably may one day (when the textural peculiarities of youth are better understood,) aid in giving a clue to its mode of action.

